# Partial Reversal of Striatal Damage by Palmitoylethanolamide Administration Following Perinatal Asphyxia

**DOI:** 10.3389/fnins.2019.01345

**Published:** 2020-01-08

**Authors:** Lucas D. Udovin, Tamara Kobiec, María I. Herrera, Nicolás Toro-Urrego, Carlos F. Kusnier, Rodolfo A. Kölliker-Frers, Ana B. Ramos-Hryb, Juan P. Luaces, Matilde Otero-Losada, Francisco Capani

**Affiliations:** ^1^Institute of Cardiological Research, University of Buenos Aires, National Research Council (ININCA-UBA-CONICET), Buenos Aires, Argentina; ^2^Centro de Investigaciones en Psicología y Psicopedagogía (CIPP), Pontificia Universidad Católica Argentina, Buenos Aires, Argentina; ^3^Departamento de Biología, Universidad Argentina John F. Kennedy (UAJK), Buenos Aires, Argentina

**Keywords:** neuroprotection, striatum, palmitoylethanolamide, perinatal asphyxia, neurofilaments

## Abstract

Perinatal asphyxia (PA) is a clinical condition brought by a birth temporary oxygen deprivation associated with long-term damage in the corpus striatum, one of the most compromised brain areas. Palmitoylethanolamide (PEA) is a neuromodulator well known for its protective effects in brain injury models, including PA, albeit not deeply studied regarding its particular effects in the corpus striatum following PA. Using Bjelke et al. (1991) PA model, full-term pregnant rats were decapitated, and uterus horns were placed in a water bath at 37°C for 19 min. One hour later, the pups were injected with PEA 10 mg/kg s.c., and placed with surrogate mothers. After 30 days, the animals were perfused, and coronal striatal sections were collected to analyze protein-level expression by Western blot and the reactive area by immunohistochemistry for neuron markers: phosphorylated neurofilament-heavy/medium-chain (pNF-H/M) and microtubule-associated protein-2 (MAP-2), and the astrocyte marker, glial fibrillary acidic protein (GFAP). Results indicated that PA produced neuronal damage and morphological changes. Asphyctic rats showed a decrease in pNF-H/M and MAP-2 reactive areas, GFAP^+^ cells number, and MAP-2 as well as pNF-H/M protein expression in the striatum. Treatment with PEA largely restored the number of GFAP^+^ cells. Most important, it ameliorated the decrease in pNF-H/M and MAP-2 reactive areas in asphyctic rats. Noticeably, PEA treatment reversed the decrease in MAP-2 protein expression and largely prevented PA-induced decrease in pNF-H/M protein expression. PA did not affect the GFAP protein level. Treatment with PEA attenuated striatal damage induced by PA, suggesting its therapeutic potential for the prevention of neurodevelopmental disorders.

## Highlights:

- Palmitoylethanolamide ameliorated neuronal damage induced by PA in rat striatum.
- Perinatal asphyxia did not alter the protein expression of GFAP in rat striatum.
- Palmitoylethanolamide largely reversed the decrease in the number of GFAP^+^ cells induced by PA in rat striatum.

## Introduction

Perinatal asphyxia (PA) is a clinical condition characterized by a transient disruption of oxygen availability, followed by metabolism deficits during birth that increases the risk of mortality and morbidity in neonates worldwide (Capani et al., 2001; Romero et al., 2015). Nearly 2–6/1,000 live births are estimated experiencing PA, rising to 10 times higher in developing countries (McGuire, 2007; Morales et al., 2011). Depending on the severity, the time elapsed from the insult, and the degree of fetal maturity, several biological processes are triggered after PA, like a decrease in the expression of synaptic proteins, cytoskeletal alterations, and protein aggregation (Herrera et al., 2017a). These events may eventually impair cellular function and lead to death and a proinflammatory response as secondary injury (Herrera et al., 2017a). Along with the hippocampus, the corpus striatum is one of the most vulnerable brain structures to PA, hypoxia (HI), and neurodegenerative injuries, partly due to its critical location in the mesolimbic and the corticostriatal circuits (Calabresi et al., 2000; Looi and Walterfang, 2013; Rocha-Ferreira and Hristova, 2016). Due to the neuronal loss and synaptic structure alterations induced by PA in these brain regions (Herrera et al., 2017a), 25% of the surviving newborns develop several neurodevelopmental abnormalities, including intellectual disability and autism spectrum disorders (Schieve et al., 2014; Modabbernia et al., 2016), attention-deficit/hyperactivity disorder (Perna and Cooper, 2012), schizophrenia (Pugliese et al., 2019), and neurodegenerative disorders (Gupta et al., 2018).

Previous studies from our group using an animal model of PA (Bjelke et al., 1991) showed that PA-induced alterations are characterized by increased astrogliosis in the hippocampus and dorsal striatum (Cebral et al., 2006; Saraceno et al., 2010; Holubiec et al., 2017), reduced microtubule-associated protein 2 (MAP-2) level, and alterations in the phosphorylation of high- (200 kDa) and medium- (160 kDa) molecular-weight neurofilaments (pNF-H/M) in the hippocampus in 120-day-old rats (P120, postnatal day 120) (Saraceno et al., 2010) or 30-day-old rats (P30, postnatal day 30) (Herrera et al., 2018). Importantly, MAP-2 is predominantly localized in neuronal dendrites of the caudate, hippocampus, cerebral cortex, and cerebellum of rodents, and its absence or diminished expression is considered as a marker of neurodegenerative disorders (Popp et al., 2009; Saraceno et al., 2012a; Romero et al., 2017). Neurofilaments (NFs) are highly phosphorylated proteins relevant to neuronal structure and function. Changes in phosphorylation alter their antigenicity and are considered a pathological hallmark of several neurodegenerative diseases (Mink and Johnston, 2000). Another biological landmark of neurodegeneration is the glial fibrillary acidic protein (GFAP), abundantly present in astrocytes in the entire central nervous system. Astroglial cells respond to brain injury and other neurological perturbations undergoing “reactive astrogliosis,” a process whereby they develop cellular hypertrophy with a concomitant increase in GFAP expression, and glial cell proliferation, indicative of neuroinflammatory processes involved in several neurodegenerative diseases (Villapol et al., 2008; Yang and Wang, 2015). There is an urgent need to improve our understanding of PA, which has currently no effective treatment and to advance in the development of effective pharmacological therapies based on specific biological targets.

One month after a severe, 20-min PA, the rats developed an alteration in the endocannabinoid system expression and related acylethanolamide pathways (Blanco et al., 2015). Palmitoylethanolamide (PEA) is an endogenous lipidic neuromodulator related to endocannabinoids, acting as a peroxisome proliferator-activated receptor alpha (PPARα) or G protein-coupled receptor 55 (GPR55) agonist that elicits anti-inflammatory, neuroprotective, and restorative properties (Mattace Raso et al., 2014; Petrosino and Di Marzo, 2017) *in vitro* (Avagliano et al., 2016), in animal models of both neurodegeneration (Esposito et al., 2011, 2012, 2014; Herrera et al., 2016) and hypoxia-ischemia (HI) (Fernandez-Lopez et al., 2013; England et al., 2015), and in human patients as well (Orefice et al., 2016). Interestingly, one study reported that natives from Puno at about 3,830 m above sea level in Peru, having high hemoglobinemia and low oxygen saturation, also exhibited a high plasma concentration of PEA (Alarcon-Yaquetto et al., 2017). This suggests that PEA might be involved in long-term adaptation to chronic high-altitude hypoxia, likely preventing the development of chronic mountain sickness (Alarcon-Yaquetto et al., 2017). Compared with other acylethanolamides, PEA showed stronger anti-inflammatory effects reducing PA-induced astrogliosis in P30 rats’ hippocampus (Holubiec et al., 2018) and preventing cortical neurons from HI-induced cell death (Portavella et al., 2018). Moreover, PEA treatment during the first hour of life in asphyctic P30 rats reduced hippocampal histological degeneration and prevented the anxiety-like behavior induced by PA (Herrera et al., 2018). However, the protective effects of PEA in the striatum of asphyctic rats have not been addressed. We hypothesized that PEA might counteract PA-induced neuronal and glial striatal damage in P30 rats. Therefore, this work aimed to investigate: (i) whether it is possible detecting neuronal cytoskeletal and glial alterations in the striatum of asphyctic P30 rats and (ii) if PEA administration within the first hour of life could ameliorate these alterations in P30 rats.

## Materials and Methods

### Animals

Sprague Dawley pregnant rats (mothers, *n* = 20) were obtained from the central vivarium at the School of Veterinary Sciences, University of Buenos Aires. Rats were maintained at 21 ± 2°C and 65% ± 5% air humidity with free access to food and water, under a 12:12-h light/dark cycle (lights on 7:00 a.m.). Fifty-three pups (and 20 mothers) were used in the study. Each animal was used only once, and all efforts were made to minimize the suffering of the animals and reduce the number of animals used. All procedures were performed by the National Institute of Health Guide for the Care and Use of Laboratory Animals (Animal Welfare Assurance, A-3033-01/protocol #S01084) and were approved by the Ethics Committee of our Institution (CICUAL #4091/04).

### Induction of PA

Fifty-three male rat pups were subjected to PA using an experimental model (Bjelke et al., 1991), which produces moderate to severe PA (Van de Berg et al., 2003; Galeano et al., 2011) as we described previously (Capani et al., 2003, 2009; Saraceno et al., 2010, 2012a,b; Blanco et al., 2015; Holubiec et al., 2017; Herrera et al., 2018). The murine model of PA, originally developed by Bjelke et al. (1991), has been widely used (Barkhuizen et al., 2017; Herrera et al., 2017b) and accepted to mimic this condition (Capani et al., 1997, 2003, 2009; Boksa and El-Khodor, 2003; Saraceno et al., 2010, 2012a,b; Strackx et al., 2010; Muñiz et al., 2014; Romero et al., 2015; Wakuda et al., 2015; Herrera et al., 2018). The Bjelke model presents several advantages such as follows: (a) asphyxia is produced at birth (Capani et al., 2009); (b) acidosis, hypercapnia, and hypoxia are present in the whole body, replicating a global asphyxia, which is the most common type (Strackx et al., 2010); (c) it is non-invasive, avoiding the effects of surgical procedures; and (d) since hypoxia is produced in the whole body, it affects both brain hemispheres, which makes this model more suitable for behavioral studies (Arteni et al., 2010). The control group was represented by non-fostered vaginally delivered pups, mimicking the clinical situation (Herrera et al., 2018).

### Neuroprotection Protocol

Fifty-three male rat pups received the respective treatment within the first hour of life. PEA (0879/10, Tocris Bioscience, Bristol, United Kingdom) was dissolved in vehicle (VHI), a solution containing 1:1:8 dimethyl sulfoxide, Tween 80, and NaCl and administered by subcutaneous injection in a volume of 10 mg/kg (Herrera et al., 2018). This dose has previously shown to be effective in animal models of experimental spinal cord injury (Genovese et al., 2008), 1-methyl-4-phenyl-1,2,3,6-tetrahydropyridine (MPTP)-inducedmodel of Parkinson’s disease (Esposito et al., 2012), lipopolysaccharide-induced neuroinflammation (Sayd et al., 2014), hippocampal damage and behavioral disturbances induced by PA (Herrera et al., 2018), and cognitive function impairment and astrogliosis induced by neonatal anoxia/ischemia in rats (Holubiec et al., 2018). A preparation similar to that used in this study inhibited the response of mesolimbic dopamine neurons to nicotine in the ventral tegmental area, suggesting the ability of PEA to cross the blood-brain barrier in rats (Melis et al., 2008). Control groups received the respective vehicle (VHI). The different groups were marked and mixed in litters with a surrogate mother. After weaning, rats were housed in cages of three to four animals from the same group. Cesarean controls were not used since previous studies revealed that this group did not present significant differences with vaginal controls (Galeano et al., 2011; Blanco et al., 2015). The selection of male animals is supported by the fact that estrogens exert neuroprotective properties (Saraceno et al., 2010). Four experimental groups were studied: rats subjected to PA and injected with VHI (PA–VHI group, *n* = 15), rats born vaginally (controls–CTL) and injected with VHI (CTL–VHI group, *n* = 13), rats subjected to PA and injected with PEA (PA+PEA group, *n* = 18), and rats born vaginally and injected with PEA (CTL+PEA group, *n* = 7). Four animals were used as controls each for immunolabeling and WB assays.

### Tissue Fixation and Immunohistochemistry

We evaluated PA-induced dorsal striatal damage in rats (*n* = 16) at P30. This age fairly resembles 4–11 human years (Semple et al., 2013), when the first signs of neurodevelopmental disorders appear (Herrera-Marschitz et al., 2014). We studied the dorsal neostriatum for its high sensitivity to hypoxic injury focusing on the axon bundles found either in the matrix or the striosome (Capani et al., 1997; Gerfen, 1989). Four animals per group (four replicates) were used. Three sections were immunostained for each analyzed brain. Rats were anesthetized with ketamine 40 mg/kg i.p. and xylazine 5 mg/kg i.p. and intracardially perfused with 4% paraformaldehyde in a 0.1 M phosphate buffer (pH 7.4) (Saraceno et al., 2016). The brains were immediately removed, post-fixed in the 4% paraformaldehyde in 0.1 M phosphate buffer (pH 7.4) solution at room temperature for 2 h, and immersed in a 0.1 M phosphate buffer (pH 7.4) overnight at 4°C. Coronal dorsal striatum sections (sections at the coronal plane, 50 μm thick) were cut using a Vibratome (VT 1000 S, Leica Microsystems, Wetzlar, Germany). This well-set technique using a 50-micron-thick vibratome section avoids embedding the tissue in paraffin with the resulting decrease in the antigenicity, gaining label specificity.

Immunohistochemistry was performed on free-floating sections under moderate shaking. Before staining, the sections were incubated 10 min in 3% hydrogen peroxide to quench endogenous peroxidases. After three washing steps in 0.1 M phosphate buffer (pH 7.4), non-specific antibody binding sites were blocked with using 0.3% normal goat serum. Free-floating sections were incubated overnight at 4°C with anti-MAP-2 (1:250, polyclonal rabbit-IgG; ab32454, Abcam, Cambridge, United Kingdom); anti-pNF-H/M, which recognizes the KSP repeats of the phosphorylated form (1:500, monoclonal mouse-IgG; MAB1592, Millipore, Burlington, VT, United States); or anti-GFAP (1:200, monoclonal rabbit-IgG; Cell-Marque, Sigma-Aldrich Company, Code. EP672Y). After several washes, sections were incubated for 1 h at room temperature with secondary antibodies (biotinylated anti-mouse-IgG, 1:500, BA9200, or biotinylated anti-rabbit-IgG, 1:500, BA-1000; Vector, Burlingame, VT, United States). The streptavidin/horseradish peroxidase detection system (1:500, K0609; Dako, Santa Clara, CA, United States) was used for antigen staining according to the manufacturer’s recommendations. Sections were incubated with the substrate diaminobenzidine (D3939; Sigma-Aldrich Company, St. Louis, MO, United States) for 2 min at room temperature. Light microscopic images were obtained using a Leica microscope (Herrera et al., 2018).

### Morphometric Analysis

The reactive area for pNF-H/M and MAP-2 was estimated using the Image J program (Image J 1.41o; NIH, United States) as described previously, and expressed as a percentage (Herrera et al., 2018). One hundred fifty by 150 μm was sampled in each photo to estimate the percentage of reactive area for pNF-H/M and MAP-2 using the Image J program (Image J 1.41o; NIH, United States). We analyzed the dorsal striatum because it is one of the most affected areas by PA insult (Holubiec et al., 2017). The number of GFAP immunoreactive astrocytes was manually estimated in the dorsal striatum. Eighty counting frames were assessed per animal. A blind observer selected five fields for each sector from three sections of the dorsal striatum. Triplicates were performed to estimate the percentage of the reactive area and the number of GFAP immunoreactive astrocytes. The NFs and microtubules were expressed as the percentage of the relative area since they cannot be individually counted. Conversely, GFAP was expressed as the absolute value since GFAP labeling allows for visualization per astrocyte and can be counted.

### Western Blot

Western blot analysis was performed using four animals per treatment (*n* = 16, four replicates) by triplicate per brain. The rats were euthanized by decapitation, and the corpus striatum was dissected and homogenized in ice-cold lysis buffer, containing 10 mM Tris–HCl at pH 7.4, 10 mM NaCl, 3 mM MgCl_2_, 0.1% Triton X-100, and protease inhibitors. Tissues were thawed on ice and centrifuged at 14,000 rpm for 15 min at 4°C. The protein concentration was analyzed using the Bradford solution (500-0201; Bio-Rad, Richmond, CA, United States) and bovine serum albumin as the standard (Herrera et al., 2018). Total protein (80 μg) was diluted in buffer [0.3 M Tris–HCl (pH 7), 5% glycerol, 5% SDS, 1 mM EDTA, 0.1% bromophenol blue] and subjected to sodium dodecyl sulfate-polyacrylamide gel electrophoresis. Polyvinylidene difluoride membranes containing the transferred proteins were blocked with 5% non-fat milk powder and 1% bovine serum albumin in a Tris-buffered saline solution containing 0.05% Tween 20. The membranes were incubated overnight at 4°C with the following primary antibodies: anti-MAP-2 (1:1,000, polyclonal rabbit-IgG; ab32454, Abcam, Cambridge, United Kingdom), anti-PNF-H/M (1:500, monoclonal mouse-IgG; MAB1592, Millipore, Burlington, VT, United States), or anti-GFAP (1:1,000, monoclonal mouse-IgG; SC-33673, Santa Cruz Biotechnology), and anti-glyceraldehyde-3-phosphate dehydrogenase (1:3,000, rabbit-IgG, G9545; Sigma-Aldrich Company, St. Louis, MO, United States) as the loading control. Next, the membranes were incubated with rabbit and mouse, secondary horseradish peroxidase-conjugated antibodies (1:3,000, 170-6515, and 170-6516, respectively; Bio-Rad, Richmond, CA, United States) for 1 h at room temperature. The protein bands were detected using an enhanced chemiluminescence Western blotting analysis system (clarity Western enhanced chemiluminescence substrate, 1705061; Bio-Rad, Richmond, CA, United States). The films were scanned, and the optical density of protein bands was quantified using Gel-Pro Analyzer 3.1.00.00 (Media Cybernetics).

### Statistical Analysis

Results are expressed as means ± SEM. Normal distribution was checked using the Shapiro–Wilk test. The data were submitted to a two-way ANOVA. Factors were the birth condition (PA or CTL) and treatment (PEA or VHI). When birth condition-treatment interaction was significant, multiple comparisons were performed using Tukey’s *t*-test. A *p* < 0.05 was conventionally set as the significance level. Statistical analysis was carried out using GraphPad Prism^TM^ 7 software.

## Results

### Effect of PEA Treatment on PA-Induced Alterations in pNF-H/M Tissue Immunostaining and Protein Expression at P30

**FIGURE 1:**
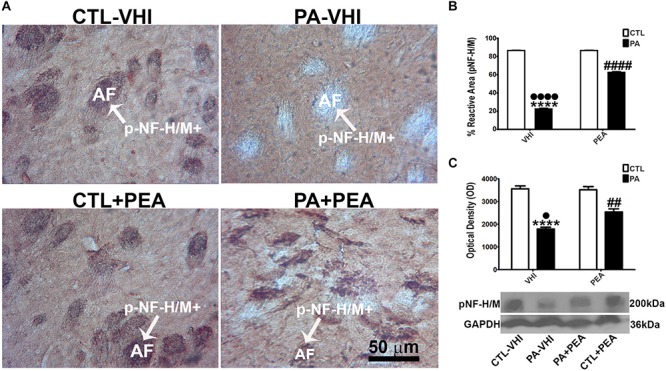
Phosphorylated neurofilament-heavy/medium-chain (pNF-H/M) immunostaining and protein level in the rat striatum in the experimental groups. **(A)** Representative images of dorsal striatum immunostained for pNF-H/M. The white arrow indicates the positive immunostaining area for pNF-H/M; AF indicates the axonal fascicles. Strictly dorsal striatum was used for immunohistochemistry due to its high vulnerability to hypoxia. Scale bar: 50 μm. **(B)** Percentage of pNF-H/M reactive area. **(C)** Optical density of bands showing pNF-H/M protein expression level. Bars and error bars represent the mean ± SEM. Two-way ANOVA followed by Tukey’s *post hoc* test. ^∗∗∗∗^*p* < 0.0001, PA–VHI vs. CTL–VHI, ••••*p* < 0.0001, and •*p* < 0.05, PA–VHI vs. PA+PEA; ####*p* < 0.0001 and ##*p* < 0.005, PA+PEA vs. CTL–VHI. PEA did not affect either pNF-H/M immunostaining or protein expression as there was no difference between CTL+PEA and CTL–VHI groups. CTL–VHI, control rats treated with vehicle; PA–VHI, rats subjected to PA and treated with vehicle; PA+PEA, rats subjected to PA and PEA treatment; CTL+PEA, control rats treated with PEA; PEA, palmitoylethanolamide.

The phosphorylation of pNF-H/M, a measure of axonal dysfunction and degeneration, was analyzed by immunostaining at P30 in the rat dorsal striatum. Figure 1A shows PA-induced morphological changes in a representative striatal section immunostained for pNF-H/M. The percentage of reactive area for pNF-H/M was dependent on birth condition and treatment [*F*_(__1_,_8__)_ = 74,552, *p* < 0.0001; *F*_(__1_,_8__)_ = 15,608, *p* < 0.0001, respectively], showing between-interaction [*F*_(__1_,_8__)_ = 15,639, *p* < 0.0001]. *Post hoc* analysis revealed that the reactive area for pNF-H/M decreased due to PA (PA–VHI) (*p* < 0.0001, Figure 1B), and that it was largely yet not completely attenuated by PEA treatment (PA+PEA) (*p* < 0.0001, Figure 1B). Consistently, at P30, striatal protein expression for pNF-H/M was dependent on birth condition and treatment [*F*_(__1_,_8__)_ = 122.3, *p* < 0.0001; *F*_(__1_,_8__)_ = 8.002, *p* = 0.0222, respectively], also showing between-interaction [*F*_(__1_,_8__)_ = 10.18, *p* = 0.0128]. *Post hoc* analysis confirmed a decrease in pNF-H/M protein expression in the PA–VHI group (*p* < 0.0001), largely yet not completely ameliorated by PEA treatment (PA+PEA) (*p* = 0.0119, Figure 1C) (*p* = 0.0024, Figure 1C). Finally, pNF-H/M reactive area and protein level were not different between the control group treated with either vehicle or PEA [CTL–VHI vs. CTL+PEA, *p* = 0.9997 (reactive area); *p* > 0.9937 (protein level); Figures 1B,C]. For more detail, please see Supplementary Figures 1 and 2.

### Effects of PEA Treatment in Dendritic Cytoskeleton Alterations and MAP-2 Tissue Immunostaining and Protein Expression at P30 After PA

**FIGURE 2:**
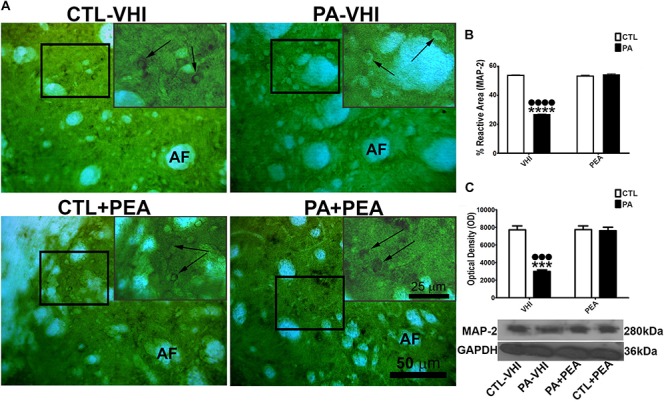
Microtubule-associated protein (MAP-2) immunostaining and protein level in the rat striatum in the experimental groups. **(A)** Representative images of the dorsal striatum immunostained for MAP-2. The figure shows MAP-2 immunostaining in the dorsal striatum; AF indicates the axonal fascicles. Scale bar: 50 μm. The main-image marked rectangular areas are shown magnified in the corresponding upper right margin. The black arrow indicates the positive immunostaining area for MAP-2. Scale bar: 25 μm. **(B)** Percentage of MAP-2 reactive area. **(C)** Optical density of bands showing for MAP-2 protein expression level. Bars and error bars represent the mean ± SEM. Two-way ANOVA followed by Tukey’s *post hoc* test. ^∗∗∗∗^*p* < 0.0001 and ^∗∗∗^*p* < 0.001, PA–VHI vs. CTL–VHI; ••••*p* < 0.0001 and •••*p* < 0.001, PA–VHI vs. PA+PEA. PEA had no effect on either MAP-2 immunostaining or protein expression level as there was no difference between CTL+PEA and CTL–VHI groups. No difference was found in MAP-2 immunostaining or protein expression level between PA+PEA and CTL–VHI groups. CTL–VHI, control rats treated with vehicle; PA–VHI, rats subjected to PA and treated with vehicle; PA+PEA, rats subjected to PA and PEA treatment; CTL+PEA, control rats treated with PEA.

Since PA affected neuronal cytoskeleton, we also studied morphological changes through immunostaining of the dendrite-specific marker MAP-2 in the rat dorsal striatum at P30. Morphological changes were observed due to PA (PA–VHI) (Figure 2A). The MAP-2 reactive area was dependent on both birth condition and treatment [*F*_(__1_,_8__)_ = 16,629, *p* < 0.0001; *F*_(__1_,_8__)_ = 17,810, *p* < 0.0001, respectively]. Birth condition-treatment interaction was also significant [*F*_(__1_,_8__)_ = 17,711, *p* < 0.0001]. *Post hoc* analysis revealed a decrease in the percentage of MAP-2 reactive area in the PA–VHI group compared with the control group (CTL–VHI) (*p* < 0.0001, Figure 2B), which was fully reversed by PEA treatment (PA+PEA) (*p* < 0.0001, Figure 2B) reaching control values (*p* = 0.0515 for reactive area; *p* = 0.9974 for protein level, Figure 2C). The analysis of MAP-2 protein expression in the rat striatum at P30 confirmed these findings. The protein level of MAP-2 was dependent on birth condition and treatment [*F*_(__1_,_8__)_ = 40.14, *p* = 0.0002; *F*_(__1_,_8__)_ = 36.83, *p* = 0.0003, respectively], with significant between-interaction [*F*_(__1_,_8__)_ = 36.09, *p* = 0.0003]. The *post hoc* analysis confirmed that PA (PA–VHI group) decreased MAP-2 protein level (*p* < 0.001, Figure 2C), which was fully restored to control values by PEA treatment (PA+PEA) (*p* < 0.001, Figure 2C), reaching control values (*p* = 0.9974, Figure 2C). Finally, MAP-2 reactive area protein level was not different between the control group treated with either vehicle or PEA (CTL–VIH vs. CTL+PEA *p* = 0.9932 for the reactive area, *p* > 0.9999 for protein level) (Figures 2B,C, respectively).

### GFAP Immunostaining and Protein Expression at P30 After PA: Protective Effect of PEA Treatment

**FIGURE 3:**
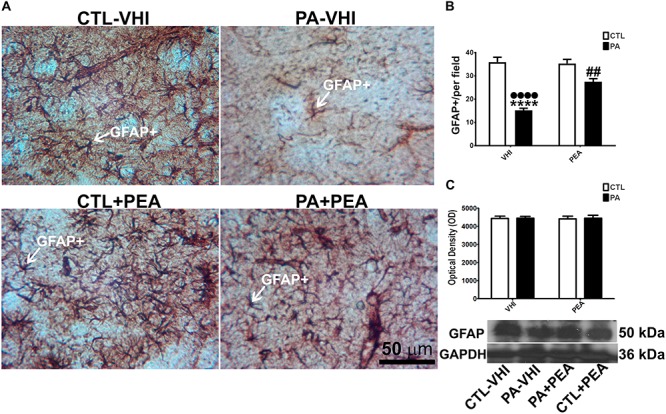
GFAP^+^ cells and protein expression level in the rat striatum. **(A)** Representative images of the dorsal striatum immunostained for glial fibrillary acidic protein (GFAP). The white arrow shows an astrocyte immunostained for GFAP. Scale bar: 50 μm. **(B)** Number of the GFAP^+^ cells measured per experimental group. **(C)** Optical density of the bands for GFAP protein expression level. Bars and error bars represent mean ± SEM. Two-way ANOVA followed by Tukey’s *post hoc* test. ^∗∗∗∗^*p* < 0.0001, PA–VHI vs. CTL–VHI, ••••*p* < 0.0001, PA–VHI vs. PA+PEA and ##*p* < 0.005, PA+PEA vs. CTL–VHI. PEA had no effect on GFAP^+^ immunostaining as there was no difference between CTL+PEA and CTL–VHI groups. Neither PA nor PEA affected GFAP protein level. CTL, control rats treated with vehicle; PA–VHI, rats subjected to PA and treated with vehicle; PA+PEA, rats subjected to PA and PEA treatment; CTL+PEA, control rats treated with PEA.

We used immunohistochemistry and Western blot to analyze glial response to PA injury and PEA treatment in the rat dorsal striatum at P30 (*n* = 16). Figure 3A shows a representative striatal section immunostained for GFAP^+^ immunoreactive astrocytes. The number of GFAP^+^ immunoreactive astrocytes was dependent on birth condition and treatment [*F*_(__1_,_56__)_ = 58.73, *p* < 0.0001; *F*_(__1_,_56__)_ = 16.44, *p* = 0.0002, respectively] showing between-interaction [*F*_(__1_,_56__)_ = 19.4, *p* < 0.0001]. The *post hoc* analysis revealed that PA (PA–VHI) reduced the number of GFAP^+^ immunoreactive astrocytes compared with the control group (CTL–VHI) (*p* < 0.0001, Figure 3B). This decrease was, to some extent, reversed by PEA treatment (PA+PEA) (*p* < 0.0001, Figure 3B) without reaching control values (*p* = 0.0048, Figure 3B). Also, the number of GFAP^+^ immunoreactive astrocytes was not different between vehicle-treated (CTL–VHI) or PEA-treated control rats (CTL+PEA) (*p* = 0.9946, Figure 3B). However, neither birth condition or treatment affected GFAP expression [*F*_(__1_,_8__)_ = 0.04421, *p* = 0.8387; *F*_(__1_,_8__)_ = 0.01157, *p* = 0.917, respectively] nor between-interaction was observed [*F*_(__1_,_8__)_ = 0.002636, *p* = 0.9603, Figure 3C].

## Discussion

Present findings show PA-induced morphological alterations like dendritic damage (MAP-2 reduction), axonal injury (pNF-H/M reduction), and a decrease in the number of astrocytes (GFAP^+^ cells) in the dorsal corpus striatum at P30. The effects on both MAP-2 and pNF-H/M were confirmed by Western blot. The MAP-2 is a commonly used biomarker to assess the extension and distribution of dendritic cytoskeletal degeneration induced by hypoxia-ischemia (HI) (Mink and Johnston, 2000; Kühn et al., 2005; Graham et al., 2018). Immunoreactivity for MAP-2 decreased in the human post-mortem hippocampus and cerebral cortex after carbon monoxide (CO) exposure (Kühn et al., 2005). Transient or permanent middle cerebral artery occlusion followed by reperfusion reduced MAP-2 expression mainly in rat striatum and neocortex (Popp et al., 2009). Also, MAP-2 immunostaining decreased in the cerebral cortex, caudate, and thalamus in a perinatal stroke rat model (Malinak and Silverstein, 1996), and in the cerebral cortex of neonatal rats after transient cerebral HI (Blomgren et al., 1995). In our study, PA induced a marked decrease both in protein level and immunoreactivity of MAP-2 in rat striatum at P30. In agreement, previous studies from our laboratory reported a significant reduction of MAP-2 at P30 in the striatum (Saraceno et al., 2012a). Similar results were found in the hippocampus at the same time point (Herrera et al., 2018), and later at P120 (Saraceno et al., 2010). This reduction of MAP-2 could be related to a decrease of adenosine triphosphate synthesis triggered by the low availability of oxygen after the hypoxic event (Islam and Burns, 1980).

This study also shows a substantial decrease in protein level and immunoreactivity of pNF-H/M in the corpus striatum in response to PA at P30 that might result from calpain, a calcium-dependent protease largely distributed in the brain, activation (Schlaepfer, 1987) by hypoxia (Stys and Jiang, 2002). Likewise, a decrease in volume and a loss of phosphorylated NFs were found in the corpus callosum and the internal capsule in experimental cerebral palsy (Drobyshevsky et al., 2007). Although we have recently demonstrated that PA was associated with an increase in protein level and pNF-H/M immunoreactivity in the CA1 hippocampus at P30 (Herrera et al., 2018), the level of pNF-H varies depending on the brain region, being low in the cerebral cortex and intermediate to high in the large-diameter brain stem axons, cerebellum, thalamus, subcortical white matter, and basal ganglia, as well as in varied pathological conditions (Shaw et al., 2005; Drobyshevsky et al., 2007; Anderson et al., 2008; Guy et al., 2008; Lewis et al., 2008; Boylan et al., 2009; Herrera et al., 2018). Bearing this in mind, the choice of different tissue sources may account for the discrepancies between studies, making further research necessary.

Unlike the consistency found for either pNF-H/M or MAP-2, discrepant findings were observed between a change in GFAP measured as the percentage of the reactive area (immunohistochemistry and the lack of change in optical density in the amount of protein (Western blot). The difference in the anatomical region used in each case might well account for the discrepancy. The dorsal striatum, the most vulnerable area to hypoxia, was used for immunohistochemical determinations. Conversely, due to methodological issues, the entire corpus striatum, both ventral and dorsal, was used for Western blot analysis, likely what might dilute any changes that might have eventually been observed otherwise. On top of this, negligible differences might have gone undetected due to insufficient sensitivity in the Western blot determinations.

Notwithstanding that GFAP did not appear as an early marker of hippocampal damage after PA, we assessed the number of GFAP^+^ astrocytes in the dorsal striatum based on our previously reported PA-induced changes in GFAP immunostaining in this region at P30 (Holubiec et al., 2017). Presently, PA induced a remarkable decrease in the number of astroglial cells. This finding likely attributable to astrocytic death might precede neuronal loss and inflammatory reaction according to previous evidence (Villapol et al., 2008; Hostenbach et al., 2014; Romero et al., 2014). In perinatal ischemia followed by reperfusion, the proapoptotic protein Bax was up-regulated in GFAP^+^ astrocytes, particularly in the peri-infarct region in the cortex at P30 post-ischemia (Benjelloun et al., 1999; Villapol et al., 2008), and apoptotic and necrotic morphological features are seen in the same astrocytes 12–24 h after oxygen-glucose deprivation, accompanied by autophagosomes that appear in the astrocytes’ cytoplasm in both ischemic cortices (Xu and Zhang, 2011). After neonatal ischemia, these dying reactive astrocytes might participate in the ongoing deleterious process and promote the formation of cystic lesions as clinically observed in the newborn brain (Villapol et al., 2008). However, our results in GFAP immunostaining were not confirmed by Western blot. Therefore, further studies are necessary to clarify this point.

Treatment with PEA (10 mg/kg) within the first hour of life attenuated PA-induced dendritic and axonal cytoskeletal damage in the dorsal striatum at P30 according to the change in MAP-2 and pNF-H/M both immunostaining and protein levels, respectively. In addition, PEA treatment prevented the decrease in the number of GFAP^+^ cells in the PA dorsal striatum at P30. Recently, we reported that PEA neuroprotected from dendritic and axonal cytoskeletal alterations induced by PA in the hippocampus (Herrera et al., 2018). Interestingly, Blanco et al. (2015) showed that PA induced a substantial decrease in the protein level of the PEA receptor, PPARα, and in N-acyl phosphatidylethanolamine-specific phospholipase D (Blanco et al., 2015), an enzyme that synthesizes acylethanolamides like PEA (Harrison et al., 2014) also at P30. This reduction could lead to dysregulation and a decrease in endocannabinoids level, inhibiting PPARα activity (Blanco et al., 2015). Then, reasonably enough, the exogenous administration of PEA may be responsible for the protective effects reported in this study.

Furthermore, our findings agree with previous reports on the protective effects of PEA in experimental neurodegeneration (Skaper et al., 1996; Esposito et al., 2012; Scuderi et al., 2014; Herrera et al., 2016). PEA protected from the decrease in MAP-2 expression induced by amyloid peptides (Scuderi et al., 2014; Tomasini et al., 2015) and by MPTP in an animal model of Parkinson’s disease (Esposito et al., 2012). Similarly, PEA could prevent MAP-2 reduction after hypoxia (Kühn et al., 2005; Fernandez-Lopez et al., 2013; England et al., 2015).

All in all, the experimental evidence supports the protective role of PEA against neuronal damage in several brain injuries, including PA-induced brain damage. Consistently with a vast number of studies, the present work reinforces the evidence of the neuroprotective effects of PEA against HI in the corpus striatum, a vulnerable brain structure to HI injuries, fostering our further investigation of the mechanisms involved.

A next study should examine the ultrastructural effects of PEA treatment regarding its presently reported effects on the expression of pNF-H/M and MAP-2, ruling out any possible bias in the interpretation of optical microscopy images.

## Data Availability Statement

All datasets generated for this study are included in the article/Supplementary Material.

## Ethics Statement

The animal study was reviewed and approved by the National Institute of Health Guide for the Care and Use of Laboratory Animals, School of Medicine, University of Buenos Aires.

## Author Contributions

LU and AR-H: acquisition, analysis, and interpretation of the data and drafting the work. TK and MH: interpretation of the data, bibliographic research, and drafting the work. NT-U, CK, RK-F, and JL: acquisition, analysis, and interpretation of the data. MO-L: critical revision for intellectual content and substantive editing, grammar and language style. FC: conception and design of the work and supervision.

## Conflict of Interest

The authors declare that the research was conducted in the absence of any commercial or financial relationships that could be construed as a potential conflict of interest.

## Abbreviations

ANOVA: analysis of variance
CNS: central nervous system
CO: carbon monoxide
GFAP: glial fibrillary acidic protein
HI: hypoxia-ischemia
HIE: hypoxia-ischemia encephalopathy
MAP-2: microtubule-associated protein-2
MPTP: 1-methyl-4-phenyl-1,2,3,6-tetrahydropyridine
PEA: palmitoylethanolamide
PA: perinatal asphyxia
PPAR α: peroxisome proliferator-activated receptor alpha
pNF-H/M: phosphorylated high/medium-molecular-weight NF.
