# Supplementary material for: Partial Reversal of Striatal Damage by Palmitoylethanolamide Administration Following Perinatal Asphyxia
Source: Front Neurosci. 2020 Jan 8;13:1345. doi: 10.3389/fnins.2019.01345 (PMC6960201; doi:10.3389/fnins.2019.01345)
Supplement: Supplementary file 1 [file Data_Sheet_1.pdf]

Supplementary Material

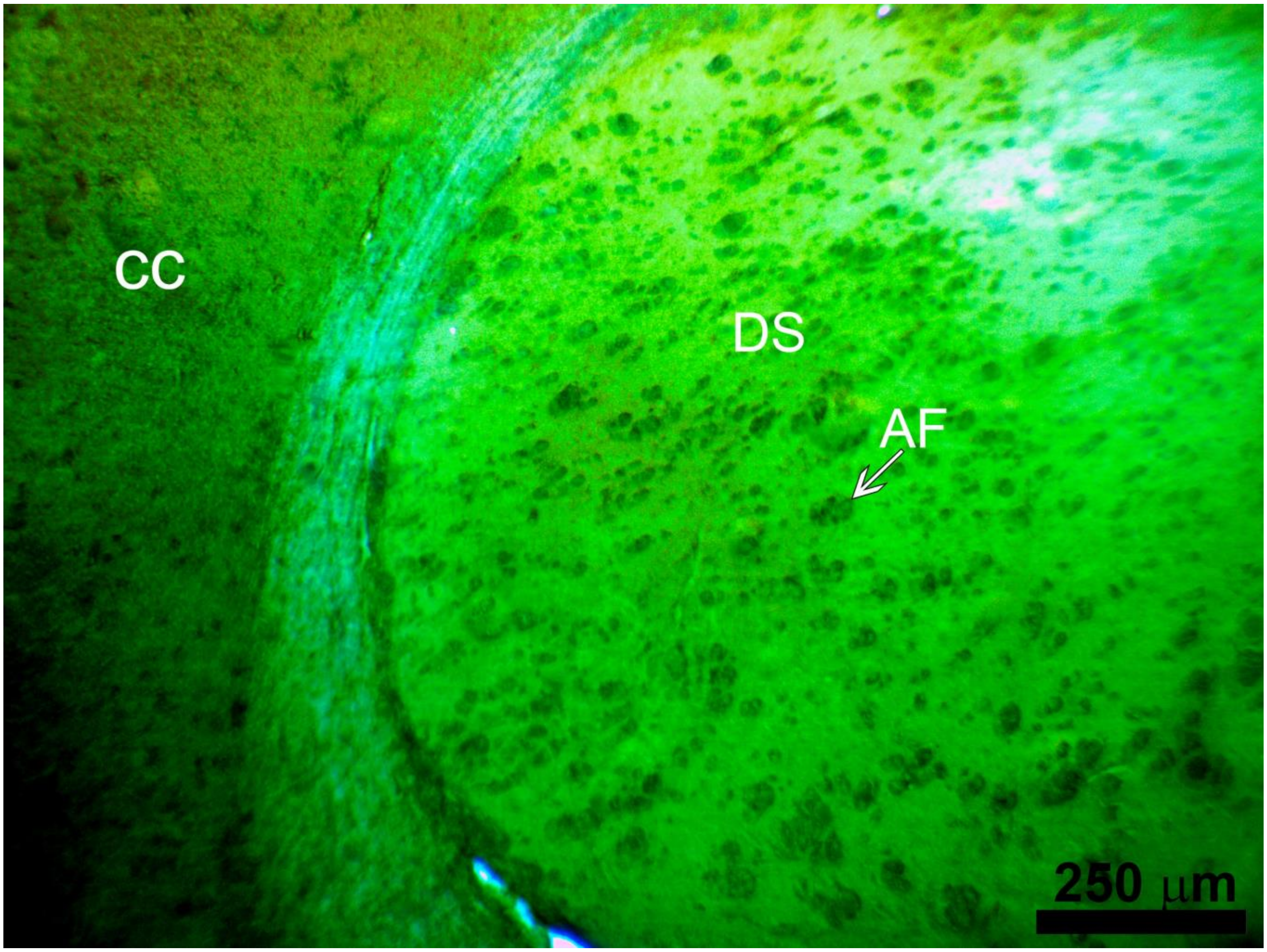

Supplementary Figure 1. General neurofilament staining of the measured reactive area region

The black arrow indicates the overall dorsal striatum staining area, and the white arrow indicates the specific axonal fascicles' reactive area. AF: axonal fascicles. CC: cerebral cortex. DS: dorsal striatum. Scale bar: 250 µm.

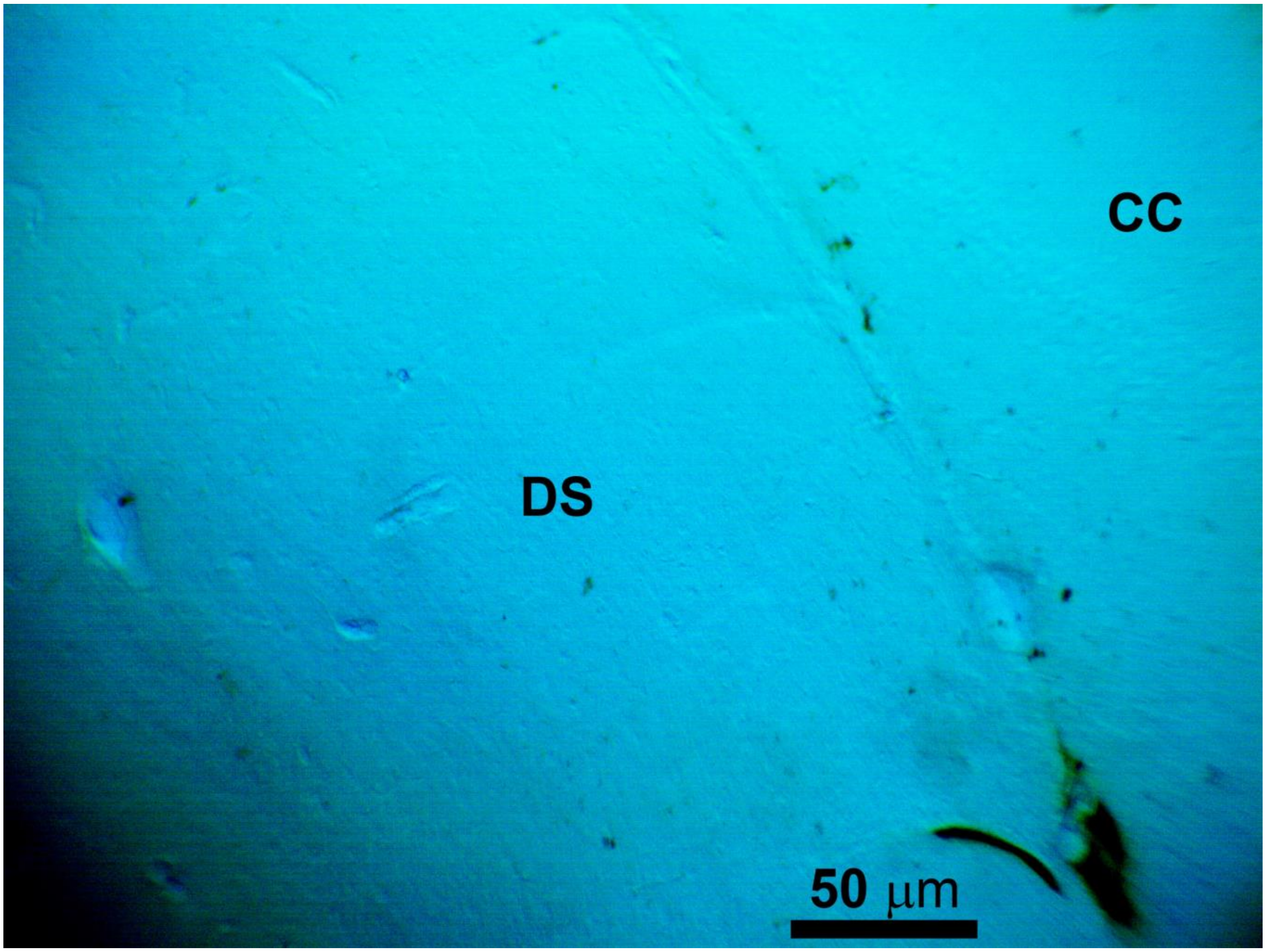

Supplementary Figure 2. Negative neurofilament staining control

Negative controls using secondary antibodies were obtained for all reactive area measures. AF: axonal fascicles. CC: cerebral cortex. DS: dorsal striatum. Scale bar: 50 μm.
